# Effects of Independent Component Analysis on Magnetoencephalography Source Localization in Pre-surgical Frontal Lobe Epilepsy Patients

**DOI:** 10.3389/fneur.2020.00479

**Published:** 2020-06-02

**Authors:** Giovanni Pellegrino, Min Xu, Abdulla Alkuwaiti, Manuel Porras-Bettancourt, Ghada Abbas, Jean-Marc Lina, Christophe Grova, Eliane Kobayashi

**Affiliations:** ^1^Neurology and Neurosurgery Department, Montreal Neurological Institute, McGill University, Montreal, QC, Canada; ^2^Department of Geriatrics, Zhongnan Hospital of Wuhan University, Wuhan, China; ^3^Multimodal Functional Imaging Laboratory, Biomedical Engineering Department, McGill University, Montreal, QC, Canada; ^4^Département de Génie Électrique, École de Technologie Supérieure, Montreal, QC, Canada; ^5^Centre de Recherches Mathematiques, Univeristé de Montréal, Montreal, QC, Canada; ^6^Physics Department and PERFORM Centre, Concordia University, Montreal, QC, Canada

**Keywords:** interictal epileptiform discharges, magnetoencephalography, magnetic source imaging, spike, independent component analysis, frontal epilepsy, MEG, source localization

## Abstract

**Objective:** Magnetoencephalography source imaging (MSI) of interictal epileptiform discharges (IED) is a useful presurgical tool in the evaluation of drug-resistant frontal lobe epilepsy (FLE) patients. Yet, failures in MSI can arise related to artifacts and to interference of background activity. Independent component analysis (ICA) is a popular denoising procedure but its clinical application remains challenging, as the selection of multiple independent components (IC) is controversial, operator dependent, and time consuming. We evaluated whether selecting only one IC of interest based on its similarity with the average IED field improves MSI in FLE.

**Methods:** MSI was performed with the equivalent current dipole (ECD) technique and two distributed magnetic source imaging (dMSI) approaches: minimum norm estimate (MNE) and coherent Maximum Entropy on the Mean (cMEM). MSI accuracy was evaluated under three conditions: (1) ICA of continuous data (Cont_ICA), (2) ICA at the time of IED (IED_ICA), and (3) without ICA (No_ICA). Localization performance was quantitatively measured as actual distance of the source maximum in relation to the focus (Dmin), and spatial dispersion (SD) for dMSI.

**Results:** After ICA, ECD Dmin did not change significantly (*p* > 0.200). For both dMSI techniques, ICA application worsened the source localization accuracy. We observed a worsening of both MNE Dmin (*p* < 0.05, consistently) and MNE SD (*p* < 0.001, consistently) for both ICA approaches. A similar behaviour was observed for cMEM, for which, however, Cont_ICA seemed less detrimental.

**Conclusion:** We demonstrated that a simplified ICA approach selecting one IC of interest in combination with distributed magnetic source imaging can be detrimental. More complex approaches may provide better results but would be rather difficult to apply in real-world clinical setting. In a broader perspective, caution should be taken in applying ICA for source localization of interictal activity. To ensure optimal and useful results, effort should focus on acquiring good quality data, minimizing artifacts, and determining optimal candidacy for MEG, rather than counting on data cleaning techniques.

## Introduction

About 30% of patients affected by focal epilepsy are drug-resistant and may be considered for surgical candidacy. Epilepsy surgery success is however conditional to the accurate identification and resection of the cortical region generating epileptiform activity. This is relatively easier to achieve in well-defined epileptic syndromes such as temporal lobe epilepsy with mesial temporal sclerosis, with up to 84% seizure freedom at one year post-surgery (1). Identification of the focus is far more complex in patients with extratemporal epilepsy and especially frontal lobe epilepsy (FLE). The latter constitutes the second most frequent surgery group following mesial temporal lobe resections, but seizure freedom rates are still disappointing at ~50% at one year post-surgery (2). One crucial question is the accuracy we achieve in localizing the generator within the frontal lobes.

Magnetoencephalography (MEG) can be a valuable neuroimaging tool to localize non-invasively the frontal epileptic focus and to guide intracranial electroencephalography (iEEG) planning as well as cortical resections (3–5). MEG signals capture the magnetic field of the epileptiform activity and allow the identification of its generator through the estimation of a forward model and the solution of the inverse problem (entire process known as source localization and/or magnetic source imaging-MSI) (6–10). The median MSI accuracy is very high with MEG source being usually within a few millimeters from the actual focus (11). In many cases, however, this procedure fails, and the generator is not accurately localized (12). Source localization failure may be related to multiple aspects along the chain of data acquisition and analysis, including, head movements in the scanner, few spikes recorded, artifacts of multiple types, inaccuracy of MRI, or MEG-MRI co-registration, inaccuracy of the forward, or inverse models, and much more (12). Most often, the reason for inaccurate source localization is the interference of noise (ocular, muscular, EKG, movement artifacts) and background activity (prominent alpha, sleep features at the time of IEDs, etc), which affects the spike field and the reconstructed source in a rather unpredictable way.

In FLE patients, it is often not trivial to recognize a misplaced generator and causes of MSI failure. Frequently, however, low signal-to-noise ratio, presence of artifacts and interference of background activity play a major role.

All these biases could be in principle identified and compensated for using techniques for data denoising (13). Among these techniques, Independent Component Analysis (ICA) (14)—a multivariate linear decomposition approach—is very popular and versatile, and it is in principle effective for cleaning any type of artifact except for that resulting from motion (15–19). The assumption behind ICA is that the activity measured -here with MEG- results from the linear combination of signals generated from multiple independent sources within the brain as well as from other origins (for example muscular and ocular activity). ICA decomposition, therefore, can unmix these signals in a number of maximally independent components (IC). The ICs untangled by ICA correspond to spatial filters, which identify statistically independent sources of MEG activity. Re-combining the entire set of ICs allows retrieving the original signal, whereas selecting/rejecting ICs allows selecting/rejecting brain sources/other activity. Neuroscientific research and experimental studies of source localization of epileptiform activity suggest some benefits of these procedures on source imaging accuracy (20–27).

The literature, however, lacks studies assessing the potential benefit and limitations of ICA denoising for interictal MEG signals source localization in clinical practice. Therefore, guidelines derived from current literature also lack a clear guidance on this topic. According to the American Clinical Magnetoencephalography Society, despite the potential usefulness of principal component analysis and ICA to estimate the reasonable number of sources in the signal above background noise and to remove ECG and eye artifacts, they are not recommended as routine techniques (28).

The recent IFCN-endorsed practical guidelines for clinical MEG highlight that ICA is computationally demanding and that the selection of IC might be problematic, operator dependent, and time consuming (13). The choice of the ICs set to retain (i.e., corresponding to epileptiform activity) and to discard (i.e., related to non-desired physiological/artifactual origin) is arbitrary. Each IC combination results in a different reconstructed signal and consequently in a different MEG source configuration. In clinical setting, where the goal is to localize the generator of epileptiform activity, this procedure becomes cumbersome.

In this study we aimed to evaluate whether selecting only one IC of interest based on its similarity with the average IED field improves source localization in patients with drug-resistant FLE.

## Materials and Methods

### Patients

The study was designed and conducted in agreement with the Declaration of Helsinki and its later amendments, approved by the Research Ethics Board of the Montreal Neurological Institute and Hospital—McGill University Health Center. All patients signed a written informed consent prior to participation in the study. We retrospectively studied a cohort of 17 patients with medically refractory FLE that underwent a MEG recording session. All patients were recruited at the Montreal Neurological Institute and Hospital and underwent a full pre-surgical evaluation with identification of the epileptic focus. We included all patients with a minimum of 5 MEG IEDs during the recording and whose MRI did not show extensive cortical lesions that could potentially hamper estimation of the forward model.

The epileptic focus of each patient was delineated on the basis of the combination of iEEG (whenever available), epileptogenic MRI lesions and extension of cortex resected during surgery, as previously described in (6, 7, 11). Two epileptologists (GP and EK) manually drew the presumed clinical epileptic focus on the cortical surface extracted from the individual anatomical high-resolution MRI. The identification of the epileptic focus was deemed accurate and the patient included when at least one of the following was satisfied: (1) patient underwent surgery and became seizure free; (2) invasive EEG capturing interictal and/or ictal activity; (3) epileptogenic MRI lesion concordant with scalp EEG findings (11) [Pellegrino et al., 2020—in press].

We compared three source localization conditions: (1) no data cleaning (No_ICA), (2) ICA on continuous data (Cont_ICA), and (3) ICA applied on short epochs time-locked to the IED (IED_ICA). The effects of ICA were quantitatively estimated with some of the most popular approaches for source localization in epilepsy (11): (1) the *equivalent current dipole (ECD)* (29, 30), (2) the *Minimum Norm Estimate (MNE)*-a linear distributed MSI (dMSI) approach (31, 32), and (3) the *coherent maximum entropy on the mean (cMEM)*—a non-linear dMSI approach developed for EEG-MEG imaging of IED (11, 33, 34). Demographic and clinical features of our cohort are reported in Table 1.

**Table 1:** Epidemiological and clinical features.

**ID**	**Age range**	**EEG**		**iEEG**			**MRI findings**	**Surgery**	**Engel class**
		**IEDs**	**Ictal**	**electrodes/Side:Regions**	**Interictal**	**Ictal**			
PA01	15–20	Bil F	Bil F				R F gyration abnormality		
PA02	15–20	Bil C	Bil C	3/R: RAC,SMA,Lesion	Lesion	Lesion	R F parasagittal FCD	R F	1
PA03	15–20	Bil FC (L>R)	Bil F	5/R:OF,Ca,Cm,SMAa,SMAp; 5/L: OF,Ca,Cm,SMAa,SMAp	bil F (L> R)	Bil F, max L SMA		L F	2
PA04	20–25	BiF F (L>R)	L F	4/R: OF,Ca,Cp,Lesion; 4/L: OF,Ca,Cp,F	Lesion, Bil F	Bil F, max Lesion	L Fa FCD, parasagittal.	L F FCD	4
PA05	20–25	RF					R F FCD		
PA06	30–35	Bil F (R>L)	RF	9/R: OF,Ca,Cm,SMAa,SMAp,Ia,Ip,A; 1/L:Hc	R OF,Ia	R OF,Ia	R hemimegalencephally	R OF	4
PA07	20–25	R FT	RF	9/R: A,Ha,Hp,Im,OF,Ca,Cm,SMAa,SMAp	OF, F convexity, Ta neocortex	OF	R F FCD	R OF	1
PA08	40–45	R FC	R FC	9/R: A,Ha,Hp,Ip,OF,SMA,Ca,Cp,P	H,SMA,Cm,OF	T,SMA		R F	3
PA09	20–25	RF	RF	2/L: H,Ip; 7/R: H,Ip,SMAa,SMAm,SMAp,Ca,Cp	R SMAa,SMAm,SMAp	R SMA	R F FCD	RF	3
PA10	220–25	Bil F	Bil F (R>L)	7/R: H,OF,Fp,Ca,Cm,SMAa,SMAp; 2/L:OF,Ca	Bil F (R>L);	Bil F (R>L)	R F FCD		
PA11	35–40	RC	R FC	2/L: SMAa,SMAp; 6/R: H,I,Ca,Cp,SMAa,SMAp	R SMAp	R SMAp	R FC parasagittal FCD		
PA12	30–35	Bil FC	R FC	7/R:A,H,OF,Ca,Cm,Ia,Ip	Fm	Fm	R Fm FCD	R Fm	1
PA13	35–40	R FT	R FT	8/R: Fa,OF,Ca,Cp,SMAa,SMAp,A,H	OF	OF	R OF FCD	R OF	1
PA14	25–30	L FC	L FC				R F polar FCD	R F polar	1
PA15	15–20	L FT	L FT				L F (opercular) FCD		
PA16	25–30	L FC	L F				L F (precentral) FCD		
PA17	25–30	Bil F (L>R)	L F				L Ca FCD	L F	1

*L, Left; R, Right; Bil, Bilateral; F, Frontal; C, Central; P, Parietal; T, Temporal; O, Occipital; a, Anterior; m, middle; p, Posterior; Post Quad, Posterior Quadrant; SMA, Supplementary Motor Area; C, Cingulate; H, Hippocampus; A, Amigdala; I, Insula; OF, Orbito Frontal; FCD, Focal cortical dysplasia; MTS, Mesial Temporal Sclerosis*.

### ICA Strategy

We considered the ICA implementation known as “runica,” which is freely offered in the EEGlab toolbox (35) and corresponds to the infomax ICA method (14). ICA was applied at sensor level prior to source localization. Prior to ICA, MEG data was filtered and detrended (see further) to meet the assumption of covariance stationarity.

We considered two different strategies: (1) ICA on continuous data, regardless of the time of IEDs occurrence, and (2) ICA taking into account only short epochs lasting 2 s and centered around the IEDs. For both approaches 20 components were extracted (36). We opted for applying an efficient, user-friendly approach for IC selection, keeping only one IC of interest, and discarding the remaining 19. The retained IC was identified visually by a trained neurophysiologist (GP) as the one showing a topographical distribution similar to the average spike field and at the same time presenting the lowest noise and background activity level. IEDs were marked according to American MEG Society guidelines, i.e., IEDs showing a dipolar spike field (28). To be noted, it is typically assumed that good (non-artifactual) ICs are dipolar (37–40) (Figure S1).

Overall, our approach was meant to: (1) allow a rapid and practical IC selection, (2) retain the most representative spatial features of the IEDs, and (3) discard both artifactual and background activity which are of no interest in the context of localizing the epileptic focus. Ultimately, we compared the accuracy of source localization without ICA (*No_ICA*), with that of *Cont_ICA* and *IED_ICA*.

### MEG Data Acquisition and Pre-processing

MEG signals were acquired with a CTF MEG system (MISL, Vancouver, Canada) installed in a 3-layer passive magnetically shielded room made by NKP (NKK Plant Engineering Corporation) of Yokohama, Japan. Patients were comfortably lying down in a supine position. All acquisitions lasted about one hour and were divided in blocks of 6 min. Data was acquired from 275 axial gradiometers. Additional bipolar electrodes were used to record electrocardiogram and electrooculogram. All patients were acquired at 1,200 Hz. The position of the head in the dewar was continuously monitored with three localization coils installed on anatomical landmarks (left and right preauricular points and nasion) and detected by the continuous head localization system of the MEG scanner. The positions of the anatomic landmarks and at least 200 evenly spaced head points were digitized using a Polhemus system for MEG-MRI co-registration. Data processing was performed in Matlab environment (The Mathworks, version 2017b), with the Brainstorm toolbox, a freely available software dedicated to the analysis of MEG and other neurophysiology data (41).

Data pre-processing consisted of the following steps: (1) spatial gradient noise cancellation of third order; (2) DC offset correction; (3) band-pass filtering (0.3–70 Hz); (4) notch filtering (60 Hz); (5) resampling to 600 Hz; (6) visual inspection and marking of the IEDs at their peak; (7) identification of epochs (duration 2 seconds, from −1 second to +1 second, being 0 the IED peak); (8) Average of epochs belonging to the same run and type (42–44). Each IED average corresponded to a source imaging “study,” as it was characterized by a specific time course, topographical distribution, head model (6, 7, 11, 45–48).

### Anatomical MRI, Forward Model

A high-resolution anatomical MRI was acquired for all patients (T1W MPRAGE sequence with the following parameters: 1 mm isotropic 3D images, 192 sagittal slices, 256 × 256 matrix, TE 52.98 ms, TR 52.3 s). Brain segmentation and cortical reconstruction were performed with FreeSurfer (http://surfer.nmr.mgh.harvard.edu/ version 6.00) (49, 50). The anatomical MRI and the cortical mesh corresponding to the layer equidistant from the white/gray matter and pia (also called “mid” layer) were then imported into the Brainstorm toolbox. Here the cortical mesh was downsampled to 8,000 vertices and the skull surface was computed. The relative position of the brain MRI and MEG sensors was obtained thanks to a MEG-MRI coregistration procedure based on surface fitting operated with a rigid geometrical transformation (three rotations, three translations) between the head shape obtained from the MRI and the fiducials and head points digitized at every MEG scan. For dMSI, we computed a 1-layer Boundary Element Method (BEM) method (51) as implemented in the OpenMEEG toolbox (52). The source model consisted in dipolar sources on every vertex of the mesh, oriented perpendicular to the cortical surface. For ECD, we computed an overlapping spheres head model (11, 28, 53). In this case the source space was not constrained to the cortical surface and the dipole could be found at any depth in the brain. For both models we assumed the conductivity of the inner-skull surface to be 0.33 S/m.

### Inverse Problem

The effect of ICA was tested on three different inverse solution techniques (Figure S2). For all methods, source localization was performed within a time window of 10 ms around the peak of the IED. Noise-covariance was modeled from a 1 s baseline without any visually identified IEDs. The baseline was extracted from the selected IC for *Cont_ICA* and *IED_ICA*. A diagonal covariance matrix was considered for all three inverse methods evaluated in this study.

#### Equivalent Current Dipole Technique

For ECD, single dipoles were fitted considering all MEG channels and without any a priori definition of the initialization point. Dipole fitting was based on the freely-available routine of Fieldtrip (54), as implemented in Brainstorm (41).

#### Distributed Magnetic Source Imaging With MNE

Minimum Norm Estimate (MNE) (32) is a linear approach, which finds sources explaining the measurements while imposing a constraint of minimum energy on the resulting source map, which is equivalent to Tikhonov regularization to solve an under-determined linear problem (i.e., MNE minimizes the L2-norm of the current distribution). MNE implementation in Brainstorm software was considered here.

#### Distributed Magnetic Source Imaging With cMEM

MEM is a non-linear distributed source imaging method based on a probabilistic Bayesian approach and on maximization of relative entropy for regularization purposes (33). The prior knowledge is incorporated in a so-called reference distribution, which relies on the assumption that brain activity is organized in cortical parcels. Cortical parcellation is performed based on MEG data, with a data driven approach (Data Driven Parcellation, DDP) (55). To this aim, a pre-localization of the sources of brain activity is performed through a multivariate source pre-localization (56). The latter is a projection method assessing for each source its possible contribution to the data. The parcellation is then obtained with a region-growing algorithm around the local maxima of the MSP map. Each parcel is characterized by a hidden state variable, assessing its probability to be active or not. The prior probability of the state of the parcel (active or not), is initialized using the median of MSP coefficients within each parcel. The method also allows creating a contrast of current intensity within active parcels. Altogether, MEM allows switching off cortical parcels that do not contribute to the solution. In this study we applied the coherent version of MEM (cMEM), which imposes an additional spatial smoothness of the solution within each parcel as proposed and evaluated in (34). cMEM was developed to localize the cortical source of IEDs with high contrast between the generator and the surrounding regions, being sensitive to its spatial extent along the cortical surface. All MEM approaches are freely available online in an add-on package of the brainstorm software “Brain Entropy in space and time (BEst) plugin” (http://neuroimage.usc.edu/brainstorm/Tutorials/TutBEst/). Further details on the mathematical aspects and validations of this method can be found in (8, 34, 47, 57, 58).

### Performance and Spatial Properties Source Imaging

**Figure 1:**
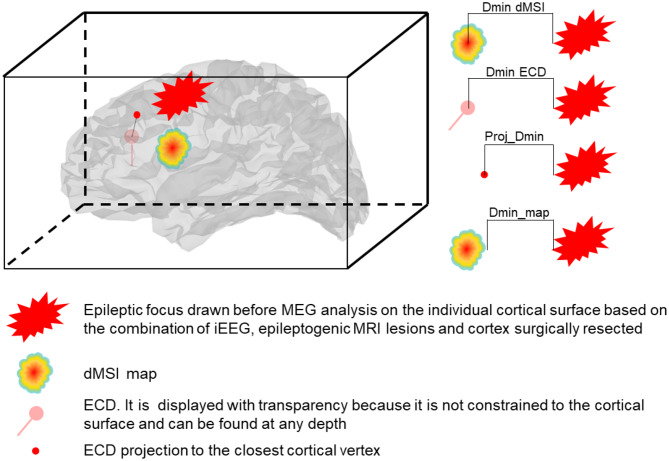
Representation of the measures of accuracy. Dmin is the Euclidean distance expressed in mm between the source and the focus. Dmin dMSI is the distance between the maximum intensity (one single vertex) cortical map and the closest point of the focus. Dmin ECD is the distance between the ECD and the closest point of the focus. Proj_Dmin is the distance between the ECD projected to the closest cortical vertex and the closest point of the focus. Dmin_map is the distance between the border of the cortical map thresholded at 30% of the maximal intensity and the closest point of the focus.

The following quantitative metrics of localization accuracy and spatial properties were estimated (Figure 1):

1. *Dmin*. It is the Euclidean distance between the source and the epileptic focus, expressed in millimeters. When considering ECD, Dmin was computed: (a) considering the actual position of the dipole in the brain and (b) its projection to the closest cortical vertex. The latter measure was denoted as *Proj_Dmin* and was meant to control for the fact that dipoles are not constrained to the cortical surface but can be found at any depth in the brain. For dMSI, Dmin was computed considering the vertex exhibiting the largest current amplitude. We also introduced the metric *Dmin_map*, for which Dmin corresponded to the distance between dMSI map thresholded at 30% of its maximal amplitude and the presumed epileptic focus.
2. *Spatial Dispersion (SD)*. It only applies to dMSI, as ECD localizations do not provide spatially extended maps. SD provides an estimate of both spatial spread and localization error with respect to the location and extension of the epileptic focus (59). The better the source map (smaller localization error, lower activity spread beyond the focus), the lower is SD. For mathematical details about the computation of this measure the reader is referred to (6, 8, 59).

Statistical analysis was performed with Matlab (The Mathworks) and SPSS (Version 24). The effect of ICA (*No_ICA* vs *Cont_ICA* vs *IED_ICA*) was evaluated independently for each inverse solution approach. Data distribution was checked with the Kolmogorov and Smirnov test. As the distribution of several variables was not Gaussian, statistical significance was assessed by applying non-parametric tests (Friedman test, Wilcoxon Signed-Rank tests, Spearman's correlation coefficients). The significance level was set to *p* < 0.05. The alpha inflation due to multiple comparisons was compensated with Bonferroni's procedure whenever appropriate.

### Data Availability

Original data is available upon reasonable request addressed to the corresponding authors.

## Results

A total of 105 studies from 17 patients (Age 26.53 ± 8.34yo, nine males) were analyzed. Clinical details are reported in Table 1. The clinical reference (epileptic focus) was based on: MRI lesion alone (*N* = 4 patients), iEEG + MRI lesion (*N* = 2), iEEG + Surgery (*N* = 2), MRI lesion + Surgery (*N* = 2), MRI lesion + iEEG + Surgery (*N* = 7).

### Effect of ICA on ECD (Descriptive Statistics in Table 2)

**Figure 2:**
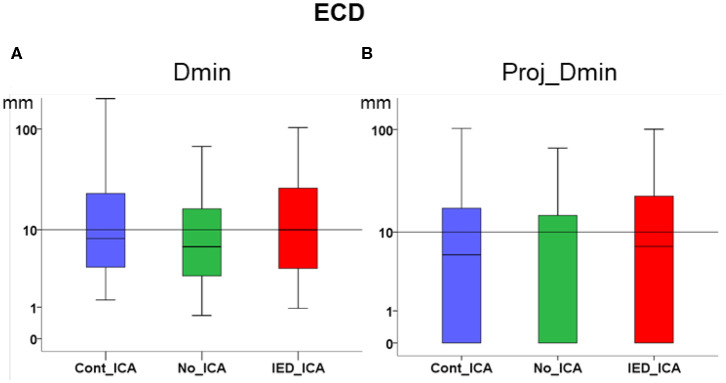
Effect of ICA on ECD. **(A)** Dmin was not significantly different across ICA approaches as compared to no_ICA. Note that the y axis is in logarithmic scale. The median for all three methods was close to or lower than 1 cm. **(B)** Dmin of the dipole projection to the closest cortical point. No significant difference was found across ICA approaches as compared to no_ICA.

ICA did not significantly change *Dmin* and *Proj_Dmin* [*Dmin* Friedman's test = 1.543, df = 2, *p* = 0.462; *Proj-Dmin* Friedman's test = 0.887, df = 2, *p* = 0.642] (Figures 2A,B).

**Table 2:** Descriptive statistics.

		**Cont_ICA**		**No_ICA**		**IED_ICA**	
		**Mean (SEM)**	**Median (Range)**	**Mean (SEM)**	**Median (Range)**	**Mean (SEM)**	**Median (Range)**
*ECD*	*Dmin*	20.40 (3.01)	8.06 (194.58)	13.59 (1.52)	6.56 (67.15)	19.38 (2.17)	9.96 (102.18)
	*Proj_Dmin*	13.29 (2.01)	5.72 (102.47)	10.82 (1.59)	0.00 (66.51)	16.25 (2.2)	7.05 (100.68)
*MNE*	*Dmin*	24.59 (2.81)	15.62 (102.79)	13.05 (2.05)	2.10 (88.13)	31.16 (2.90)	24.43 (143.54)
	*Dmin_Map*	0.37 (0.21)	0.00 (16.29)	0.14 (0.08)	0.00 (7.41)	0.32 (0.16)	0.00 (10.74)
	*SD*	47.51 (0.97)	46.68 (37.80)	40.56 (1.00)	40.36 (41.78)	52.48 (1.11)	50.09 (51.03)
*cMEM*	*Dmin*	13.01 (1.73)	6.54 (86.96)	9.85 (1.41)	4.84 (74.01)	31.60 (3.4)	23.90 (143.77)
	*Dmin_Map*	3.32 (1.12)	0.00 (76.95)	2.11 (0.71)	0.00 (45.56)	8.40 (1.71)	0.00 (74.5)
	*SD*	22.63 (1.58)	17.55 (87.99)	20.03 (1.23)	15.95 (54.01)	43.60 (2.01)	42.08 (106.88)

*Cont_ICA, ICA of continuous data; No_ICA, without ICA; IED_ICA, ICA at the time of IED; ECD, equivalent current dipole; MNE, minimum norm estimate; cMEM, coherent Maximum Entropy on the Mean; Dmin, Euclidean distance between the source and the epileptic focus, expressed in millimeters; Proj_Dmin, distance between projection of the dipole to closest cortical vertex and the epileptic focus, expressed in millimeters; Dmin_Map, Dmin computed considering dMSI maps with a 30% threshold; SD, Spatial Dispersion*.

### Effect of ICA on MNE (Descriptive Statistics in Table 2)

**Figure 3:**
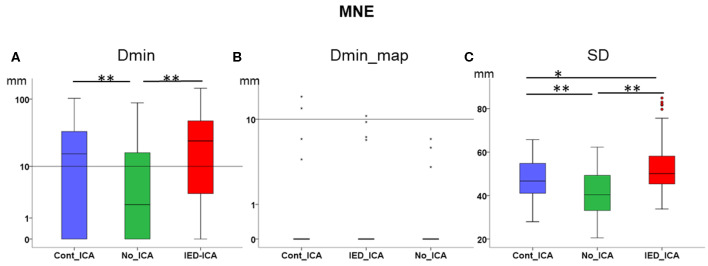
Effect of ICA on MNE. **(A)** Dmin was significantly lower (better) for No_ICA as compared to Cont_ICA and IED_ICA. **(B)** Dmin_map did not differ across approaches, probably due to a floor effect. **(C)** The spatial dispersion was significantly higher (worse) for both Cont_ICA and IED_ICA as compared with No_ICA. SD was also higher for IED_ICA as compared to Cont_ICA. *p < 0.05; **p < 0.001.

*Dmin* was significantly higher for *IED_ICA* and *Cont_ICA* as compared *No_ICA* [Friedman's test = 31.714, df = 2, *p* < 0.001; *post-hoc IED_ICA* vs. *No_ICA z* = 0.629, *p* < 0.001; *post-hoc Cont_ICA* vs. *No_ICA z* = 0.571, *p* = 0.002] (Figure 3A). Dmin_map did not differ across ICA approaches, probably because of a floor effect. SD was overall very large for MNE, lower for *No_ICA* as compared to both *IED_ICA* and *Cont_ICA*, with the latter being lower than IED_ICA [Friedman's test = 142.419, df = 2, *p* < 0.001; *post-hoc IED_ICA* vs. *No_ICA z* = 1.581, *p* < 0.001; *post-hoc Cont_ICA* vs. *No_ICA z* = 1.190, *p* < 0.001; *post-hoc Cont_ICA* vs. *IED_ICA z* = 0.390, *p* < 0.014] (Figure 3C).

### Effect of ICA on cMEM (Descriptive Statistics in Table 2)

**Figure 4:**
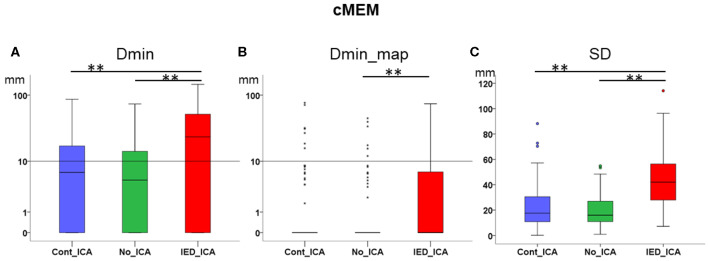
Effect of ICA on cMEM. **(A)** Dmin was significantly lower (better) for No_ICA and Cont_ICA as compared to IED_ICA. **(B)** Dmin_map was significantly worse for IED_ICA as compared to No_ICA. **(C)** The spatial dispersion was significantly higher (worse) for IED_ICA as compared with No_ICA and Cont_ICA. **p < 0.001.

*Dmin* was significantly higher for *IED_ICA* as compared to both *No_ICA* and *Cont_ICA* [Friedman's test = 26.677, df = 2, *p* < 0.001; *post-hoc IED_ICA* vs. *No_ICA z* = 0.595, *p* < 0.001; *post-hoc IED_ICA* vs. *Cont_ICA z* = 0.476, *p* = 0.002]. No significant difference was found between *Cont_ICA* and *No_ICA* [*z* = 0.119, *p* > 0.5] (Figure 4A). When looking at *Dmin* of the map *(Dmin_map)*, which takes into account the spatial extent of the generator, *No_ICA* performed better than *IED_ICA* [Friedman's test = 20.272, df = 2, *p* < 0.001; *post-hoc IED_ICA* vs. *No_ICA z* = 0.333, *p* = 0.043] (Figure 4B). In the same line, the study of SD confirmed the detrimental effect of ICA on source imaging [Friedman's test = 74.133, df = 2, *p* < 0.001] with *IED_ICA* being worse than both *No_ICA* and *Cont_ICA* [*post-hoc IED_ICA* vs. *Cont_ICA z* = 1.048, *p* < 0.001; *post-hoc IED_ICA* vs. *No_ICA z* = 1.010, *p* < 0.001] (Figure 4C).

### Effect of the Number of Averaged IEDs on Source Localization Performance

The performance of *Cont_ICA* and *IED_ICA* could be in principle influenced by the “amount of signal of interest” that we wanted to separate, i.e., by the number of IEDs which were identified. We therefore addressed the relationship between the number of IEDs averaged per study and the performance of source localization. We restricted this analysis to the following measures: *Dmin* for ECD, *Dmin* for cMEM, *Dmin* for MNE, *SD* of cMEM, and *SD* of MNE.

There was a significant relationship between the number of IEDs and source localization performance, as highlighted by all measures under investigation [*Spearmans's Rho, pooling together No_ICA, Cont_ICA and IED_ICA; Dmin ECD Rho* = −*0.409, p* < *0.001; Dmin MNE Rho* = −*332, p* < *0.001; Dmin cMEM Rho* = −*0.331, p* < *0.001; SD MNE Rho* = −*0.254, p* < *0.001; SD cMEM Rho* = −*0.155, p* = *0.006*]. This relationship remained significant when correcting for the ICA approach (*No_ICA, Cont_ICA and IED_ICA)*, suggesting that the number of IEDs did significantly influence ICA performance [*Dmin ECD R* = −*0.169, p* = *0.003; Dmin MNE R* = −*0.241, p* < *0.001; Dmin cMEM R* = −*0.181, p* = *0.001; SD MNE R* = −*0.241, p* < *0.001; SD cMEM R* = −*0.122, p* = *0.031*].

## Discussion

Both dipoles and the vertex of dMSI source maps with the largest amplitude had a median distance from the focus lower than 1 cm (Figures 2A, 3A, Table 2). These results are in agreement with previous reported literature from our group and others (3–5, 11, 60–64). Selecting one IC of interest prior to applying source localization, however, does not improve MSI in a clinical cohort of drug-resistant FLE patients.

When investigating the distribution MSI performance metric over the whole population of patients, there was no definite effect of ICA on ECD. The effect of IED_ICA was detrimental on dMSI, as demonstrated by several measures investigated in this study (Figures 2, 3).

These results are somehow unexpected, as ICA was applied to improve source localization by removing artifacts and the interference of background activity.

Previous experimental studies have demonstrated that ICA-based artifact correction may improve the spatial localization of brain activity, when selecting multiple ICs of interest or rejecting multiple artifactual ICs (20–23, 36). ICA and similar blind and semiblind separation techniques are increasingly used to separate and study brain source activities (40, 65–68). ICA can separate biological signals which have time-course maximally independent from background activity or noise (example IEDs). Each IC corresponds to a source of activity contributing to the data recorded (40). On the other hand, there is only minimal evidence suggesting a potential benefit of ICA in clinical practice. Some recent studies applied ICA to clean EEG data prior the identification of high frequency oscillations (69), or to study independent generators of posterior quadrant versus anterior spikes (70, 71). ICA was also applied to estimate the number of sources with a signal above background noise prior to EEG source localization (28, 72, 73) and this is perhaps the application to which we more often perform clinical MEG analysis (28). Wennberg and Cheyne have studied a cohort of patients with a well-defined anterolateral temporal neocortex epilepsy focus, demonstrating that neither principal component analysis nor ICA improved MEG source localization (74). In our study, we in fact found worsening of source localization, especially for dMSI. The effect size was relatively small, as the median difference of the distance between the MSI generator and the focus between No_ICA and ICA approaches was in the scale of few millimeters and no difference would have been noticed if we had estimated the accuracy as lobar or sublobar concordance, rather than relying on more refined quantitative measures (6, 12, 47, 75).

In our patients the interference of ICA with MSI is likely due to the strategy of IED marking and IC selection. Both these procedures were based on the idea that the magnetic field distribution of “good spikes” and that a “good IC” is dipolar (28, 40). While this hypothesis is probably valid in most cases, it is clearly tailored for ECD rather than dMSI. ECD relies on the assumption that the source is punctual and has a dipolar magnetic field (76), reason why this technique fails when the signal to noise ratio is low, the generator is spatially extended and the magnetic field is complex (77–79). In contrast, dMSI is more robust to low signal to noise ratio signals and more appropriate to model complex fields and their propagation pattern (11, 31).

Previous literature suggests that epileptiform activity may be captured by multiple components (23). Nonetheless a previous study on realistic simulations demonstrates that ICA can even isolate two similar epileptiform transients (one transient for IC), and the retrieved IEDs were almost identical as the simulated, especially in their spatial distributions. The remaining components were capturing background activity or artifacts only and did not show any feature of epileptiform activity (80). This strongly supports the idea of selecting one component only, while also providing a definite advantage for possible clinical application. It is anyway possible that IEDs should be rather modeled by multiple components and by selecting only one IC we might have missed meaningful and useful information (23). In such conditions, selecting only one IC would result in applying source localization in a too low dimensional sub-space, which could explain why performance decreased according to our validation metric.

IED_ICA is an approach very similar to the one applied to identify multiple sources at the time of IEDs (72, 73), but in our cohort performance proved worse than that of Cont_ICA (Figures 2, 3). Although definite conclusions on the reasons for such behaviour cannot be reached with our experimental design, it could be speculated that: (a) Cont_ICA can better capture the “epileptic network” and its localization is closer to the focus, and (b) IED_ICA might in principle be severely impacted by the amount of signal with which the procedure was fed. In this respect, we found a relationship between the number of IEDs averaged across studies and the accuracy of source imaging, yet the number of spikes did not significantly affect ICA performance. In other words, the “amount of IEDs” is more relevant to increase the signal to noise ratio and, in turn, improve source localization rather than for ICA extraction.

It might be argued that extracting a dipolar IC for dMSI might not have been fully justified. This strategy, however, was motivated by our goal to set-up a simple procedure, which could be easily translated to busy clinical routines (see Introduction).

Standard MEG source localization already requires multiple operator-dependent choices, including IEDs marking, time-point selection for MSI (peak vs. raising slope), forward model, inverse solution and much more (81). Each of these choices affects MSI and might result in a different generator. In clinical setting, where the ultimate goal is to determine the location of the IED generator from MEG data, an additional step for selection of an IC set is not feasible and not affordable for the busy clinician.

This study has several limitations. Here we will list the most relevant. Firstly, only some patients included in our cohort were operated, and only few of them achieved seizure freedom. This is concordant with the real-world scenario at other quaternary epilepsy centers (2, 82, 83), but prevents us from an accurate identification of the so-called epileptogenic zone based on surgical outcome data (84). Nonetheless, we took an extra step in carefully including only those patients for whom reliable data was available for the identification of the epileptic focus, consisting of seizure freedom, or -whenever this was not possible- invasive EEG findings or MRI epileptogenic reasons. In this study, we opted for a real-world clinical definition of an “epileptic focus” which, in agreement with previous studies, took into consideration all available pre-surgical information [(6, 11, 47, 48, 75), Pellegrino et al., 2020 -in press]. Secondly, when designing the study, we opted for not focusing on individual artifacts captured by ICs, because such approach would not be feasible in clinical practice: (a) there is no consensus on how artifactual components are to be identified; (b) there is no consensus on how many components should be retained/rejected; (c) testing multiple choices of IC retained/rejected would be time-consuming and aleatory. We therefore opted for selecting only one component based on a simple criterion, so that if this procedure would be confirmed useful, the process could be later automated and easily translated to clinical practice. We acknowledge, however, that a different strategy of component selection would have brought different results, perhaps better. Thirdly, we opted for having only one operator selecting the component because we designed this work as a real-world study. It is indeed very unlikely that multiple operators may have the time/resources/opportunity to double check spikes and components in busy clinical routines. Fourthly, we focused our attention on neocortical frontal patients, who are those for whom source localization is more often needed and challenging. The results of these study should be generalized to other context with caution, as other groups -although in very specific setting and small number of patients - have demonstrated that ICA may successfully identify sources of activity not immediately evident in raw data, for instance in mesial temporal lobe epilepsy (36). Lastly, from a methodological perspective, we applied ICA at sensor level prior to inverting our signal to find the source of IEDs. ICA however could be either applied to recorded data at sensor level or after inversion at source level. Some studies show that source level ICA is more suitable for extracting resting state networks (85), whereas others recommend performing ICA prior to inversion (86). Here we applied the latter approach, largely to reduce the computational load for clinical application. Nonetheless it would be worth exploring alternative strategies.

Previous studies and daily clinical practice have often been performed without dedicated data cleaning procedure, but relying on good quality data (13, 28). The results of this study support this policy and the statement of the IFCN-endorsed practical guidelines for clinical MEG (13): “it is always preferable to prevent unwanted non-brain signals during data collection rather than to attempt to correct or compensate for them during data analysis.” ICA should remain an option for difficult cases, applied together with ECD by trained personnel. Based on our study, it should also be avoided in routine clinical MEG when dMSI is the choice of method.

## Data Availability Statement

The datasets generated for this study are available on request to the corresponding author.

## Ethics Statement

The studies involving human participants were reviewed and approved by Research Ethics Board of the Montreal Neurological Institute and Hospital—McGill University Health Center. The patients/participants provided their written informed consent to participate in this study.

## Author Contributions

GP, J-ML, CG, and EK: study design. GP, MX, AA, MP-B, CG, and EK: data acquisition. GP, MX, AA, J-ML, CG, and EK: data analysis. GP, CG, and EK: manuscript preparation. All: manuscript revision and approval.

## Conflict of Interest

The authors declare that the research was conducted in the absence of any commercial or financial relationships that could be construed as a potential conflict of interest.

## Supplementary Material

Figure S1 Example of IC selection. The left side of the picture shows the topographical distribution of the magnetic field at the peak of the average spike. 20 ICs were extracted (right panel). Only one component was retained (green box). The retained IC (green box) was identified visually as the one showing a topographical distribution similar to the average spike field and at the same time presenting the lowest noise and background activity level. To be noted, none of the other ICs had a dipolar distribution with a similar topography as the average IEDs field.

Figure S2 Example patient. From top to bottom, source localization without ICA (No_ICA), with ICA applied around IEDs (IED_ICA) and with ICA applied on continuous MEG data (Cont_ICA). From left to right, average IED time course, topography of the magnetic field at the peak, source localization performed with cMEM, MNE, and ECD. Cortical surface has been inflated to improve visibility (50% inflation). The green region of cMEM and MNE cortical surfaces indicates the focus. cMEM and MNE sources have been thresholded at 30% of the local maximum. ECD was not constrained to the cortical surface, which is made transparent to improve visibility.
