# Supplementary figures and images for: Effects of Independent Component Analysis on Magnetoencephalography Source Localization in Pre-surgical Frontal Lobe Epilepsy Patients
Source: Front Neurol. 2020 Jun 2;11:479. doi: 10.3389/fneur.2020.00479 (PMC7280485; doi:10.3389/fneur.2020.00479)

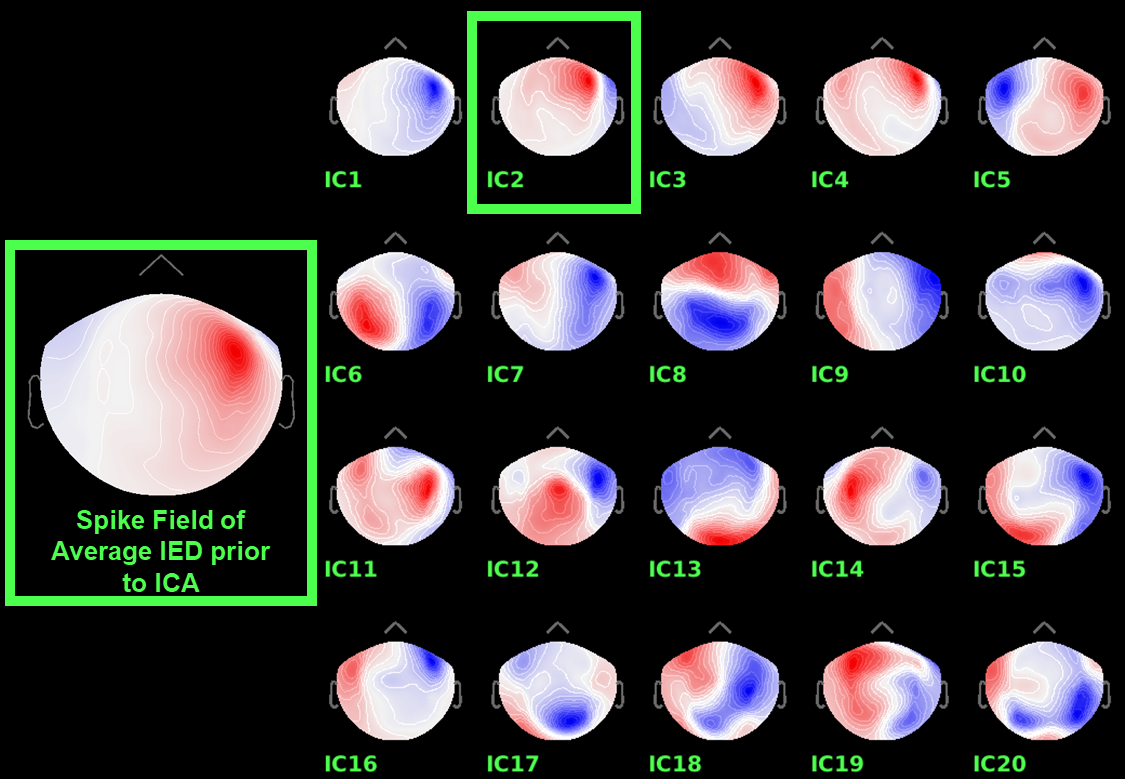

Supplement: Figure S1 — Example of IC selection. The left side of the picture shows the topographical distribution of the magnetic field at the peak of the average spike. 20 ICs were extracted (right panel). Only one component was retained (green box). The retained IC (green box) was identified visually as the one showing a topographical distribution similar to the average spike field and at the same time presenting the lowest noise and background activity level. To be noted, none of the other ICs had a dipolar distribution with a similar topography as the average IEDs field. [file Image_1.tif]

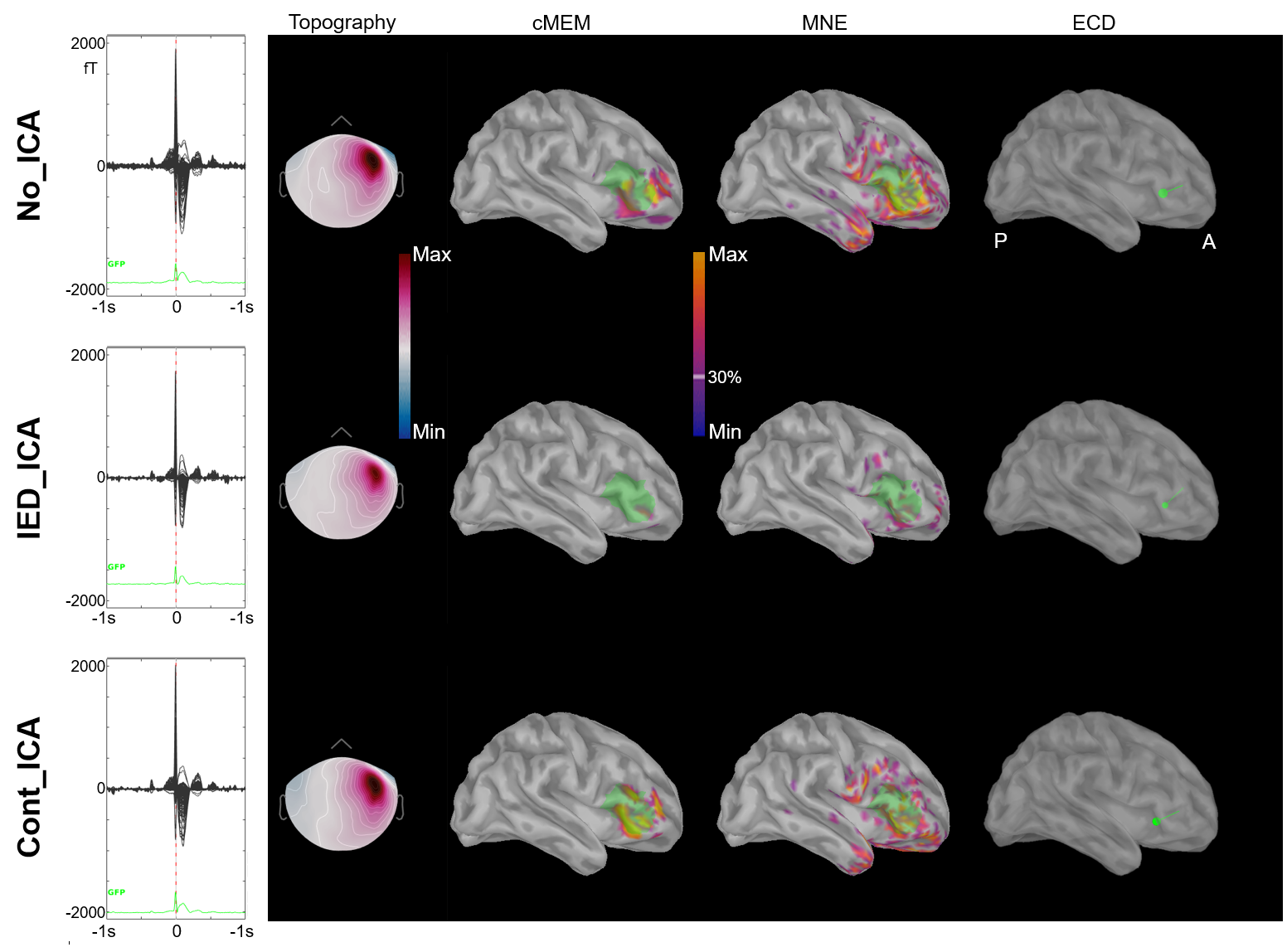

Supplement: Figure S2 — Example patient. From top to bottom, source localization without ICA (No_ICA), with ICA applied around IEDs (IED_ICA) and with ICA applied on continuous MEG data (Cont_ICA). From left to right, average IED time course, topography of the magnetic field at the peak, source localization performed with cMEM, MNE, and ECD. Cortical surface has been inflated to improve visibility (50% inflation). The green region of cMEM and MNE cortical surfaces indicates the focus. cMEM and MNE sources have been thresholded at 30% of the local maximum. ECD was not constrained to the cortical surface, which is made transparent to improve visibility. [file Image_2.tif]
